# Carbonic anhydrase activation profile of indole-based derivatives

**DOI:** 10.1080/14756366.2021.1959573

**Published:** 2021-08-02

**Authors:** Elisabetta Barresi, Rahul Ravichandran, Lorenzo Germelli, Andrea Angeli, Emma Baglini, Silvia Salerno, Anna Maria Marini, Barbara Costa, Eleonora Da Pozzo, Claudia Martini, Federico Da Settimo, Claudiu Supuran, Sandro Cosconati, Sabrina Taliani

**Affiliations:** a Department of Pharmacy, University of Pisa, Pisa, Italy; b DiSTABiF, University of Campania Luigi Vanvitelli, Caserta, Italy; c Department of NEUROFARBA, Section of Pharmaceutical and Nutraceutical Sciences, University of Florence, Firenze, Sesto Fiorentino, Italy

**Keywords:** Carbonic anhydrase activators, indole, microglia, brain associated human CA VII isoform

## Abstract

Carbonic Anhydrase Activators (CAAs) could represent a novel approach for the treatment of Alzheimer’s disease, ageing, and other conditions that require remedial achievement of spatial learning and memory therapy. Within a research project aimed at developing novel CAAs selective for certain isoforms, three series of indole-based derivatives were investigated. Enzyme activation assay on human CA I, II, VA, and VII isoforms revealed several effective micromolar activators, with promising selectivity profiles towards the brain-associated cytosolic isoform hCA VII. Molecular modelling studies suggested a theoretical model of the complex between hCA VII and the new activators and provide a possible explanation for their modulating as well as selectivity properties. Preliminary biological evaluations demonstrated that one of the most potent CAA **7** is not cytotoxic and is able to increase the release of the brain-derived neurotrophic factor (BDNF) from human microglial cells, highlighting its possible application in the treatment of CNS-related disorders.

## Introduction

Carbonic anhydrases (CAs) are metalloenzymes that play a crucial role in many physio-pathological conditions, mainly involving the maintenance/alteration of pH homeostasis^1^. In the central nervous system (CNS), CAs are involved in neuronal signalling, as the reaction they catalyse, that is the reversible hydration/dehydration of CO_2_/HCO_3_^−^ (CO_2_ + H_2_O ⇋ HCO_3_^−^ +H^+^), contributes to the availability of ions that are essential for GABAergic and glutamatergic neuronal function, such as bicarbonate and protons, able to regulate the pH transitions in both the intra- and extra-cellular compartments^2–5^. Specifically, CAs catalytic activity takes place in a reversible two-step process: (i) the conversion of CO_2_ to HCO_3_^−^, via the nucleophilic attack of a Zn-bound hydroxide on CO_2_, and the subsequent displacement of the newly formed HCO_3_^−^ by a water molecule^1^; (ii) a proton transfer process mediated by well-ordered water molecules and a histidine residue of the active site that regenerates the active enzyme featuring the Zn-bound hydroxide^1^.

$$\text{CA}-\text{Zn}\left(\text{II}\right)-{\text{OH}}^{-}+{\text{CO}}_{2}↔\text{CA}-\text{Zn}\left(\text{II}\right)-{\text{HCO}}_{3}{}^{-}+{\text{ H}}_{2}O↔\text{CA}-\text{Zn}\left(\text{II}\right)-{\text{OH}}_{2}+{\text{HCO}}_{3}{}^{-}$$

(i)


$$\text{CA}-\text{Zn}\left(\text{II}\right)-{\text{OH}}_{2}↔\text{CA}-\text{Zn}\left(\text{II}\right)-{\text{OH}}^{-}+{\text{ H}}^{+}$$

(ii)




CAs have been considered as promising targets for the development of novel small molecules with therapeutic potential for the treatment of several human diseases. While inhibitors have been widely investigated as antitumor, antiglaucoma, diuretic, anticonvulsant, and antiobesity agents, the development of activators remains a poorly exploited research field until recently^6^^,^^7^.

Recent reports highlighted that CA activation in the brain has implications for Alzheimer's disease and dementia^8^. Specifically, a role of CAs in brain processing of information and memory storage has been proposed, in terms of regulation of Cl^−^/HCO_3_^−^ potentially associated with hippocampal GABAergic postsynaptic depolarisation, that in turn correlates with the synchronised neuronal activity, called theta rhythm^9^. In addition, CA inhibition was shown to abolish theta activity, as well as antagonism at GABA_A_ receptor, and to impair learning and memory processes in rats^10^. The beneficial effects of CA activation towards enhancing memory and learning observed in ageing rat models, together with the observation that brain CA levels are diminished in age-correlated impairment of CNS functionality^11^, emphasises the importance of this family of enzymes in both disease and normal neuronal functions, demanding further development of CA activating drugs.

**Figure 1.**
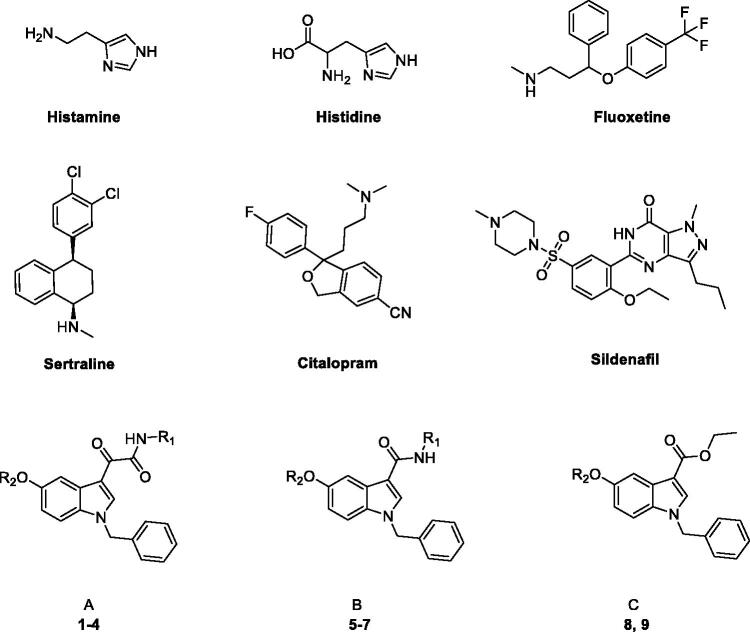
Structures of known and novel indole-based CAAs.

CA inhibitors classically feature a primary sulfonamide group that interacts with the zinc and displaces the catalytic Zn-bound-hydroxide/water necessary for the catalytic conversion of CO_2_ into HCO_3_^−^^12^. On the other hand, most classes of activators identified so far, belong to the amine, amino acid, and oligopeptide chemotypes^7^. Their mechanism, thoroughly clarified by X-ray crystallographic and kinetic studies^13–17^, basically resides in the participation in the proton transfer reaction from the water bound to the Zn^2+^ ion to the reaction medium, with the generation of the active zinc hydroxide species. This is the rate-determining step of the catalytic cycle, physiologically mediated by a His residue (His64 in many isoforms); CA activators (CAAs) function as additive proton shuttle molecules, shifting the equilibrium to the active form of the enzyme^13–17^. As few examples, phenylalanine (PDB:2FMG)^13^, histamine (PDB:1AVN)^18^, histidine (PDB:2ABE)^14^ (Figure 1), and other imidazole-based compounds^19–23^ were identified as CA activators providing an alternative or additive site for proton transfer speeding up the reaction. X-ray crystallographic studies indicated the following mechanism^13–15^^,^^18^^,^^24–26^:

$$\begin{array}{l} \text{CA}-\text{Zn}\left(\text{II}\right)-{\text{OH}}_{2}+\text{CAA}↔\left[\text{CA}-\text{Zn}\left(\text{II}\right)-{\text{OH}}_{2}-\text{CAA}\right]\\ ↔\left[\text{CA}-\text{Zn}\left(\text{II}\right)-{\text{OH}}^{-}-\text{CAA}-H^{+}\right]\\ ↔\text{CA}-\text{Zn}\left(\text{II}\right)-{\text{OH}}^{-}+\text{CAA}-H^{+} \end{array}$$





Activators bind to variable sites different from the inhibitors’ binding site and far away from the metal ion; the interested region does not necessarily overlap with that occupied by His64 situated in the middle part and extending towards the edge of the active site, but in the opposite part with respect to the active site or in an outer region^13–15^^,^^18^^,^^24–26^.

Typically, CAAs’ design was based on histidine (Figure 1), the natural proton shuttle, or histamine (Figure 1), one of the first CAAs discovered, as a lead compound and proceeded through modification of the heterocyclic scaffold (including the combinations with additional nuclei), and variable functionalization of the primary amino group also with the insertion of aromatic moieties^7^. In addition, several drugs in clinical use for various therapeutic applications, i.e. sertraline, citalopram, fluoxetine, sildenafil (Figure 1), showed CA-activating effects, which might contribute to their effects or account for some side effects^27^^,^^28^. These agents feature a molecular structure including various aromatic (poly)heterocyclic portions, more complex and not directly correlated with the leads histamine or histidine. Comprehensive SAR studies suggest a proton-shuttling group bound to a hydrophobic aromatic heterocycle by a short and flexible linker as basic structural requirements to obtain efficient CAAs. To expand the chemical space exploitable for the development of novel CAAs, in the present work, three small series of indole-based derivatives (**A**, **B**, **C**, Figure 1) were synthesised and biologically evaluated for their CA activating properties. Specifically, all the novel compounds featured the indole scaffold, a “privileged scaffold” for drug discovery^29^, decorated with a benzyl group at N1 atom of the indole ring and a protonatable moiety at different positions as a mimic of the proton shuttling residue (i.e. histidine).

In particular, the **A** series arises from our longstanding experience in the synthesis of indolglyoxylamides endowed with promising *in vivo* or *in vitro* biological activity (nonsedative/hypnotic, nonsedative/anxiolytic, antiinflammatory, neuroprotective)^30–35^, combined with the widely recognised role of the alpha-keto amide moiety as a privileged motif in medicinal chemistry^36^. In derivatives **1–4** (series **A**, Figure 1), two different protonatable (dimethylaminopropyl, diethylaminoethyl) and polar hydroxyl-decorated (hydroxypropyl, hydroxyethyl) chains were variously attached to the 5-position and the glyoxamido-nitrogen of the 1-benzylindole core. A first round of biological evaluation of compounds **1–4** on human hCA I, II, VA, and VII (vide infra) revealed a similar activation and selectivity profile for all the compounds. In line with these results, and in consideration of the synthetic feasibility, we decided to expand the SARs on these indole-based CAAs with the synthesis of compounds of series **B** and **C** (Figure 1), where the protonatable chain was maintained at 5-position of the indole, while varying the functionalization at 3-position. Specifically, derivatives **5–7** feature a 3-carboxamide moiety decorated with a hydroxyethyl (**5** and **6**) or lipophilic benzyl (**7**) group, while a simple ethyl ester moiety characterised derivatives **8** and **9** (Figure 1).

## Materials and methods

### Chemistry

The uncorrected melting points were determined using a Reichert Kofler hot-stage apparatus. ^1^H NMR and ^13 ^C NMR spectra were recorded on a Bruker AVANCE 400 (^1^H, 400 MHz, ^13 ^C, 100 MHz) in DMSO-d_6_, MeOD or CDCl_3_. Chemical shifts are expressed in *δ* (ppm) and coupling constants (J) in Hertz. Magnesium sulphate was used as the drying agent. Evaporations were made *in vacuo* (rotating evaporator). Analytical TLCs have been carried out on Merck 0.2 mm precoated silica gel aluminium sheets (60 F-254). Silica gel 60 (230–400 mesh) was used for column chromatography. Purity of the target compounds **1**–**9** was determined using a Shimadzu LC-20AD SP liquid chromatograph equipped with a DDA Detector (*λ* = 254 nm) using a column C18 (250 mm × 4.6 mm, 5 µm, Shim-pack); the mobile phase, delivered at isocratic flow, consisted of 70% of acetonitrile and 30% H_2_O (0.1% NH_3_); flow rate 1 ml/min. All the compounds showed percent purity ≥ 95%. Reagents, starting materials, and solvents were purchased from commercial suppliers and used as received. The intermediate *N*-benzyl-5-methoxy-1*H*-indole **10** was prepared according to the reported procedure^37^.

#### General procedure for the synthesis of 2-(5-(2-hydroxyethoxy)-1-benzyl-1H-indol-3-yl)-N-(dialkylaminoalkyl)glyoxylamides 1 and 2

A solution of the appropriate derivative **16** or **17** (0.31 mmol) was dissolved in 1,4-dioxane (7 ml) and cooled at 0 °C. Then a solution of NaOH 1 M (1.25 ml) was added, and the mixture was stirred at room temperature for 5 h. The reaction mixture was concentrated in vacuum, the residue dissolved in ethyl acetate and washed with saturated aqueous NaHCO_3_ solution, water and brine. The organic phase was dried over MgSO_4_ and concentrated under reduced pressure. The crude products were then purified by flash chromatography (DCM/MeOH in ratio 9:1 as eluting system).

##### 2-(5-(2-Hydroxyethoxy)-1-benzyl-1H-indol-3-yl)-N-(3-(dimethylamino)propyl)glyoxylamide (1)

Yield: 69%; oil; ^1^H NMR (400 MHz, CDCl_3_) *δ* 9.05 (s, 1H, Ar-H), 8.24 (t, *J* = 6.0 Hz, 1H, NH), 7.98 (d, *J* = 2.4 Hz, 1H, Ar-H), 7.36–7.31 (m, 3H, Ar-H), 7.20–7.18 (m, 3H, Ar-H), 6.93 (dd, *J* = 2.4 Hz, *J* = 8.8 Hz, 1H, Ar-H), 5.35 (s, 2H, CH_2_Ph), 4.21 (t, *J* = 4.4 Hz, 2H, CH_2_O), 4.00 (t, *J* = 4.4 Hz, 2H, C*H_2_*OH), 3.50–3.45 (m, 2H, C*H_2_*NH), 2.50 (t, *J* = 6.8 Hz, 2H, CH_2_N), 2.34 (s, 6H, 2CH_3_), 1.85–1.82 (m, 2H, CH_2_C*H_2_*CH_2_). ^13 ^C NMR (100 MHz, CDCl_3_) *δ* 180.23 (C=O), 162.81 (C=O), 156.04 (C-Ar) 141.59 (C-Ar), 135.39 (C-Ar), 131.53 (C-Ar), 129.04 (2 C-Ar), 128.91 (C-Ar), 128.28 (C-Ar) 126.96 (2 C-Ar), 114.19 (C-Ar), 112.16 (C-Ar), 111.51 (C-Ar), 105.63 (C-Ar), 69.90 (CH_2_O), 61.55 (CH_2_OH), 57.58 (CH_2_N), 51.43 (CH_2_Ph), 45.12 (2CH_3_), 38.11 (CH_2_NH), 26.44 (CH_2_*C*H_2_CH_2_).

##### 2-(5-(2-Hydroxyethoxy)-1-benzyl-1H-indol-3-yl)-N-(2-(diethylamino)ethyl)glyoxylamide (2)

Yield: 81%; oil; ^1^H NMR (400 MHz, DMSO-d_6_) *δ* 8.95 (s, 1H, Ar-H), 8.54 (t, *J* = 6.0 Hz, 1H, NH), 7.77 (d, *J* = 2.4 Hz, 1H, Ar-H), 7.48 (d, *J* = 8.8 Hz, 1H, Ar-H), 7.37–7.28 (m, 5H, Ar-H), 6.90 (dd, *J* = 2.4 Hz, *J* = 8.8 Hz, 1H, Ar-H), 5.55 (s, 2H, CH_2_Ph), 4.00 (t, *J* = 4.6 Hz, 2H, CH_2_O), 3.74 (t, *J* = 4.4 Hz, 2H, C*H_2_*OH), 3.30–3.23 (m, 2H, C*H_2_*NH), 2.57–2.51 (m, 6H, CH_2_N, 2C*H_2_*CH_3_), 0.97 (t, *J* = 7.2 Hz, 6H, 2CH_2_C*H_3_*). ^13 ^C NMR (100 MHz, DMSO-d_6_) *δ* 181.90 (C=O), 163.60 (C=O), 156.20 (C-Ar), 141.71 (C-Ar), 137.18 (C-Ar), 131.56 (C-Ar), 129.20 (2 C-Ar), 128.44 (C-Ar), 128.28 (C-Ar), 127.80 (2 C-Ar), 113.77 (C-Ar), 112.84 (C-Ar), 111.68 (C-Ar), 105.21 (C-Ar), 70.44 (CH_2_O), 60.12 (CH_2_OH), 51.48 (CH_2_N), 50.43 (CH_2_Ph), 47.10 (2*C*H_2_CH_3_), 37.19 (CH_2_NH), 12.37 (2CH_2_*C*H_3_).

#### General procedure for the synthesis of 2–(5-(dialkylaminoalkoxy)-1-benzyl-1H-indol-3-yl)-N-(hydroxyalkyl)glyoxamides 3 and 4, and ethyl 5-(dialkylaminoalkoxy)-1-benzyl-1H-indole-3-carboxylates 8, 9 and 22

In a flask, Cs_2_CO_3_ (62 mg, 0.19 mmol) was added to a solution of the appropriate derivative **20** or **21** or **29** (0.15 mmol) in anhydrous DMF (5 ml). In another flask, Cs_2_CO_3_ (78 mg, 0.24 mmol) was added to a suspension of the appropriate amine (3-dimethylamino-1-propylchloride hydrochloride for **3** and **8**, 2-chloro-*N,N*-diethylethylamine hydrochloride for **4** and **22**, 2-chloro-*N,N*-dimethylethylamine hydrochloride for **9**, 0.24 mmol) in 3 ml of the same solvent. The mixtures were stirred for about 30 min at room temperature and then put together and the resulting mixture was heated at 60 °C for 5 h. The solvent was removed under reduced pressure, then ice was added to the reaction mixture and the solid precipitate formed was collected by vacuum filtration. The crude products were finally purified by flash chromatography (DCM/MeOH in ratio 9:1 as eluting system).

##### 2-(5-(3-(Dimethylamino)propoxy)-1-benzyl-1H-indol-3-yl)-N-(3-hydroxypropyl)glyoxamide (3)

Yield: 56%; mp = 111–113 °C; ^1^H NMR (400 MHz, DMSO-d_6_) *δ* 8.91 (s, 1H, Ar-H), 8.68 (t, *J* = 5.6 Hz, 1H, NH), 7.76 (d, *J* = 2.8 Hz, 1H, Ar-H), 7.47 (d, *J* = 9.2 Hz, 1H, Ar-H), 7.37–7.27 (m, 5H, Ar-H), 6.89 (dd, *J* = 2.4 Hz, *J* = 8.8 Hz, 1H, Ar-H), 5.55 (s, 2H, CH_2_Ph), 4.53 (t, *J* = 5.2 Hz, 1H, CH_2_O*H*), 4.02 (t, *J* = 6.4 Hz, 2H, CH_2_O), 3.49–3.45 (m, 2H, C*H_2_*OH), 3.29–3.26 (m, 2H, C*H_2_*NH), 2.47 (t, *J* = 6.8 Hz, 2H, CH_2_N), 2.23 (s, 6H, 2CH_3_), 1.92–1.88 (m, 2H, NCH_2_C*H_2_*CH_2_O), 1.69–1.66 (m, 2H, NHCH_2_C*H_2_*CH_2_OH). ^13 ^C NMR (100 MHz, DMSO-d_6_) *δ* 182.20 (C=O), 163.87 (C=O), 156.11 (C-Ar), 141.57 (C-Ar), 137.19 (C-Ar), 131.54 (C-Ar), 129.21 (2 C-Ar), 128.41 (C-Ar), 128.27 (C-Ar), 127.80 (2 C-Ar), 113.76 (C-Ar), 112.85 (C-Ar), 111.69 (C-Ar), 105.04 (C-Ar), 66.58 (CH_2_O), 59.19 (CH_2_OH), 56.01 (CH_2_N), 50.41 (CH_2_Ph), 45.31 (2CH_3_), 36.68 (CH_2_NH), 32.42 (NHCH_2_*C*H_2_CH_2_OH), 27.07 (NCH_2_*C*H_2_CH_2_O).

##### 2-(5-(2-(Diethylamino)ethoxy)-1-benzyl-1H-indol-3-yl)-N-(2-hydroxyethyl)glyoxamide (4)

Yield: 57%; mp = 116–118 °C; ^1^H NMR (400 MHz, CDCl_3_) *δ* 9.00 (s, 1H, Ar-H), 7.97 (t, *J* = 6.0 Hz, 1H, NH), 7.94 (d, *J* = 2.4 Hz, 1H, Ar-H), 7.34–7.31 (m, 3H, Ar-H), 7.19–7.17 (m, 3H, Ar-H), 6.90 (dd, *J* = 2.4 Hz, *J* = 8.8 Hz, 1H, Ar-H), 5.34 (s, 2H, CH_2_Ph), 4.32 (t, *J* = 5.2 Hz, 2H, CH_2_O), 3.85 (t, *J* = 5.2 Hz, 2H, C*H_2_*OH), 3.60–3.56 (m, 2H, C*H_2_*NH), 3.16 (t, *J* = 5.2 Hz, 2H, CH_2_N), 2.92 (q, *J* = 6.8 Hz, 2C*H_2_*CH_3_), 1.25 (t, *J* = 6.8 Hz, 6H, 2CH_2_C*H_3_*). ^13 ^C NMR (100 MHz, CDCl_3_) δ 179.72 (C=O), 163.48 (C=O), 156.62 (C-Ar), 141.64 (C-Ar), 135.27 (C-Ar), 131.59 (C-Ar), 129.07 (2 C-Ar) 128.88 (C-Ar), 128.33 (C-Ar), 126.97 (2 C-Ar), 113.98 (C-Ar), 112.02 (C-Ar), 111.59 (C-Ar), 105.65 (C-Ar), 61.91 (CH_2_O), 51.45 (CH_2_OH), 51.10 (CH_2_N), 47.60 (CH_2_Ph), 42.23 (2*C*H_2_CH_3_), 29.69 (CH_2_NH), 10.51 (2CH_2_*C*H_3_).

##### Ethyl 5-(3-(dimethylamino)propoxy)-1-benzyl-1H-indole-3-carboxylate (8)

Yield: 88%; mp = 58–60 °C; ^1^H NMR (400 MHz, CDCl_3_) *δ* 7.81 (s, 1H, Ar-H), 7.69 (d, *J* = 2.4 Hz, 1H, Ar-H), 7.36–7.31 (m, 3H, Ar-H), 7.19–7.14 (m, 3H, Ar-H), 6.88 (dd, *J* = 2.6 Hz, *J* = 9.0 Hz, 1H, Ar-H), 5.31 (s, 2H, CH_2_Ph), 4.39 (q, *J* = 7.2 Hz, 2H, OC*H_2_*CH_3_), 4.12 (t, *J* = 6.2 Hz, 2H, OC*H_2_*CH_2_), 2.69 (t, *J* = 7.6 Hz, 2H, CH_2_N), 2.42 (s, 6H, 2CH_3_), 2.14–2.07 (m, 2H, CH_2_C*H_2_*CH_2_), 1.43 (t, *J* = 7.0 Hz, 3H, OCH_2_C*H_3_*). ^13 ^C NMR (100 MHz, CDCl_3_) *δ* 165.11 (C=O), 155.04 (C-Ar), 136.01 (C-Ar), 134.60 (C-Ar), 131.88 (C-Ar), 128.97 (2 C-Ar), 128.10 (C-Ar), 127.76 (C-Ar), 126.95 (2 C-Ar), 113.55 (C-Ar), 111.14 (C-Ar), 107.26 (C-Ar), 104.33 (C-Ar), 66.45 (O*C*H_2_CH_3_), 59.62 (O*C*H_2_CH_2_), 56.40 (CH_2_N), 50.92 (CH_2_Ph), 44.89 (2CH_3_), 26.96 (CH_2_*C*H_2_CH_2_), 14.62 (OCH_2_*C*H_3_).

##### Ethyl 5-(2-(dimethylamino)ethoxy)-1-benzyl-1H-indole-3-carboxylate (9)

Yield: 30%; mp = 69–71 °C; ^1^H NMR (400 MHz, CDCl_3_) *δ* 7.82 (s, 1H, Ar-H), 7.71 (d, *J* = 2.4 Hz, 1H, Ar-H), 7.36–7.31 (m, 3H, Ar-H), 7.19–7.14 (m, 3H, Ar-H), 6.93 (dd, *J* = 2.6 Hz, *J* = 9.0 Hz, 1H, Ar-H), 5.31 (s, 2H, CH_2_Ph), 4.39 (q, *J* = 7.2 Hz, 2H, OC*H_2_*CH_3_), 4.21 (t, *J* = 5.6 Hz, 2H, CH_2_O), 2.85 (t, *J* = 5.4 Hz, 2H, CH_2_N), 2.42 (s, 6H, 2CH_3_), 1.42 (t, *J* = 7.0 Hz, 3H, OCH_2_C*H_3_*). ^13 ^C NMR (100 MHz, CDCl_3_) *δ* 165.15 (C=O), 154.97 (C-Ar), 135.99 (C-Ar), 134.59 (C-Ar), 131.89 (C-Ar), 128.97 (2 C-Ar), 128.11 (C-Ar), 127.67 (C-Ar), 126.97 (2 C-Ar), 113.85 (C-Ar), 111.16 (C-Ar), 107.27 (C-Ar), 104.14 (C-Ar), 66.10 (O*C*H_2_CH_3_), 59.63 (O*C*H_2_CH_2_), 58.27 (CH_2_N), 50.91 (CH_2_Ph), 45.65 (2CH_3_), 14.64 (OCH_2_*C*H_3_).

##### Ethyl 5-(2-(diethylamino)ethoxy)-1-benzyl-1H-indole-3-carboxylate (22)

Yield: 42%; mp = 48–50 °C; ^1^H-NMR (400 MHz, CDCl_3_) *δ* 7.81 (s, 1H, Ar-H), 7.71 (d, *J* = 2.4 Hz, 1H, Ar-H), 7.35–7.29 (m, 3H, Ar-H), 7.18–7.13 (m, 3H, Ar-H), 6.89 (dd, *J* = 2.4 Hz, *J* = 8.8 Hz, 1H, Ar-H), 5.29 (s, 2H, CH_2_Ph), 4.39 (q, *J* = 7.2 Hz, 2H, OC*H_2_*CH_3_), 4.20 (t, *J* = 6.2 Hz, 2H, CH_2_O), 2.99 (t, *J* = 6.2 Hz, 2H, CH_2_N), 2.74 (q, *J* = 7.2 Hz, 4H, 2NC*H_2_*CH_3_), 1.42 (t, *J* = 7.0 Hz, 3H, OCH_2_C*H_3_*), 1.14 (t, *J* = 7.2 Hz, 6H, 2NCH_2_C*H_3_*).

#### General procedure for the synthesis of 5-(dialkylaminoalkoxy)-1-benzyl-N-(2-hydroxyethyl)-1H-indole-3-carboxamides 5 and 6 and 5–(2-(diethylamino)ethoxy)-N,1-dibenzyl-1H-indole-3-carboxamide 7

To an ice-cooled solution of the appropriate derivative **23** or **24** or **25** (0.25 mmol) in 5 ml of anhydrous DMF, TBTU (120 mg, 0.38 mmol) and DIPEA (87 µl, 0.50 mmol) were added and the mixture was stirred for 30 min. Then, the appropriate amine (ethanolamine for **5** and **6**, benzylamine for **7**, 0.25 mmol) was added and the resulting mixture was stirred at room temperature overnight. DMF was removed under reduced pressure and ice was added to the flask. The mixture was extracted with DCM, dried over MgSO_4_, and concentrated under reduced pressure. The crude products were purified by flash chromatography (DCM/MeOH in ratio 9:1 as eluting mixture).

##### 5-(3-(Dimethylamino)propoxy)-1-benzyl-N-(2-hydroxyethyl)-1H-indole-3-carboxamide (5)

Yield: 23%; oil; ^1^H NMR (400 MHz, MeOD) *δ* 7.88 (s, 1H, Ar-H), 7.69 (d, *J* = 2.4 Hz, 1H, Ar-H), 7.31–7.24 (m, 4H, Ar-H), 7.17–7.15 (m, 2H, Ar-H), 6.84 (dd, *J* = 2.4 Hz, *J* = 8.8 Hz, 1H, Ar-H), 5.31 (s, 2H, CH_2_Ph), 4.04 (t, *J* = 6.0 Hz, 2H, CH_2_O), 3.74 (t, *J* = 5.6 Hz, 2H, C*H_2_*OH), 3.53 (t, *J* = 5.6 Hz, 2H, C*H_2_*NH), 2.55–2.51 (m, 2H, CH_2_N), 2.28 (s, 6H, 2CH_3_), 1.99–1.93 (m, 2H, CH_2_C*H_2_*CH_2_). ^13 ^C NMR (100 MHz, MeOD) *δ* 166.98 (C=O), 154.08 (C-Ar), 137.00 (C-Ar), 132.05 (C-Ar), 131.29 (C-Ar), 128.43 (2 C-Ar), 127.50 (C-Ar), 126.78 (2 C-Ar), 112.87 (C-Ar), 111.14 (C-Ar), 109.57 (C-Ar), 103.92 (C-Ar), 60.71 (CH_2_O), 55.84 (CH_2_OH), 52.26 (CH_2_N), 50.05 (CH_2_Ph), 43.01 (2CH_3_), 41.52 (CH_2_NH), 23.01 (CH_2_*C*H_2_CH_2_).

##### 5-(2-(Dimethylamino)ethoxy)-1-benzyl-N-(2-hydroxyethyl)-1H-indole-3-carboxamide (6)

Yield: 27%; oil; ^1^H NMR (400 MHz, MeOD) *δ* 7.90 (s, 1H, Ar-H), 7.72 (d, *J* = 2.4 Hz, 1H, Ar-H), 7.32–7.26 (m, 4H, Ar-H), 7.19–7.17 (m, 2H, Ar-H), 6.89 (dd, *J* = 2.6 Hz, *J* = 9.0 Hz, 1H, Ar-H), 5.35 (s, 2H, CH_2_Ph), 4.16 (t, *J* = 5.2 Hz, 2H, CH_2_O), 3.74 (t, *J* = 5.8 Hz, 2H, C*H_2_*OH), 3.53 (t, *J* = 5.8 Hz, 2H, C*H_2_*NH), 2.83 (t, *J* = 5.2 Hz, 2H, CH_2_N), 2.38 (s, 6H, 2CH_3_). ^13 ^C NMR (100 MHz, MeOD) *δ* 166.86 (C=O), 153.10 (C-Ar), 136.96 (C-Ar), 132.41 (C-Ar), 131.55 (C-Ar), 128.44 (2 C-Ar), 127.52 (C-Ar), 126.78 (2 C-Ar), 112.67 (C-Ar), 111.41 (C-Ar), 109.67 (C-Ar), 104.21 (C-Ar), 62.32 (CH_2_O), 60.70 (CH_2_OH), 53.37 (CH_2_N), 50.10 (CH_2_Ph), 42.71 (2CH_3_), 41.54 (CH_2_NH).

##### 5-(2-(Diethylamino)ethoxy)-N,1-dibenzyl-1H-indole-3-carboxamide (7)

Yield:16%; mp= 57–59 °C; ^1^H NMR (400 MHz, MeOD) *δ* 7.99 (s, 1H, Ar-H), 7.79 (d, *J* = 2.4 Hz, 1H, Ar-H), 7.41–7.30 (m, 5H, Ar-H), 7.29–7.22 (m, 4H, Ar-H), 7.22–7.20 (m, 2H, Ar-H), 6.94 (dd, *J* = 2.6 Hz, *J* = 9.0 Hz, 1H, Ar-H), 5.42 (s, 2H, NCH_2_Ph), 4.60 (s, 2H, NHCH_2_Ph), 4.35 (t, *J* = 5.0 Hz, 2H, CH_2_O), 3.52 (t, *J* = 5.0 Hz, 2H, CH_2_N), 3.26 (q, *J* = 7.4 Hz, 4H, 2C*H_2_*CH_3_), 1.34 (t, *J* = 7.2 Hz, 6H, 2CH_2_C*H_3_*). ^13 ^C NMR (100 MHz, MeOD) *δ* 166.48 (C=O), 153.51 (C-Ar), 139.40 (C-Ar), 136.92 (C-Ar), 132.42 (C-Ar), 131.37 (C-Ar), 128.44 (2 C-Ar), 128.09 (2 C-Ar), 127.66 (C-Ar), 127.51 (C-Ar), 127.05 (2 C-Ar), 126.77 (2 C-Ar), 126.63 (C-Ar), 112.73 (C-Ar), 111.26 (C-Ar), 109.60 (C-Ar), 104.26 (C-Ar), 63.02 (CH_2_O), 51.14 (CH_2_N), 50.14 (2CH_2_Ph), 42.48 (2*C*H_2_CH_3_), 8.04 (2CH_2_*C*H_3_).

#### General procedure for the synthesis of 2-(1-benzyl-5-methoxy-1H-indol-3-yl)-N-(dialkylaminoalkyl)glyoxylamides 12 and 13

Oxalyl chloride (0.18 ml, 2.0 mmol) was added dropwise at 0 °C to a solution of *N*-benzyl-5-methoxy-1*H*-indole **10** (250 mg, 1.0 mmol) in freshly distilled diethyl ether (10 ml). The mixture was maintained at room temperature for 2 h. The generated precipitate was collected by vacuum filtration to give the acyl chloride **11** that was directly used in the subsequent reaction. A solution of the appropriate amine (*N*,*N*-dimethyl-1,3-propylenediamine for **12**, *N*,*N*-diethylethylenediamine for **13**, 0.81 mmol) in dry toluene (5 ml) was added dropwise, under a nitrogen atmosphere, to a stirred suspension, cooled at 0 °C, of 2–(1-benzyl-5-methoxy-1*H*-indol-3-yl)glyoxylyl chloride **11** (222 mg, 0.68 mmol) in 5 ml of the same solvent, followed by the addition of NEt_3_ (0.12 ml, 0.88 mmol). The reaction mixture was left under stirring at room temperature overnight. The solvent was removed under reduced pressure, and the residue was dissolved with DCM. The organic solution was washed with a 5% solution of NaHCO_3_, 10% solution of HCl, H_2_O, dried over MgSO_4_ and evaporated to dryness, yielding the desired compounds **12**, **13** that were used in the subsequent step without further purification.

##### 2-(1-Benzyl-5-methoxy-1H-indol-3-yl)-N-(3-(dimethylamino)propyl)glyoxylamide (12)

Yield: 48%; mp = 186–188 °C; ^1^H NMR (400 MHz, DMSO-d_6_) *δ* 8.93 (s, 1H, Ar-H), 8.89 (t, *J* = 6.0 Hz, 1H, NH), 7.78 (d, *J* = 2.8 Hz, 1H, Ar-H), 7.51 (d, *J* = 8.8 Hz, 1H, Ar-H), 7.38–7.28 (m, 5H, Ar-H), 6.92 (dd, *J* = 2.4 Hz, *J* = 8.8 Hz, 1H, Ar-H), 5.57 (s, 2H, CH_2_Ph), 3.80 (s, 3H, OCH_3_), 3.31–3.28 (m, 2H, C*H_2_*NH), 3.07–3.03 (m, 2H, CH_2_N), 2.73 (s, 6H, 2CH_3_), 1.94–1.90 (m, 2H, CH_2_C*H_2_*CH_2_).

##### 2-(1-Benzyl-5-methoxy-1H-indol-3-yl)-N-(2-(diethylamino)ethyl)glyoxylamide (13)

Yield: 77%; mp = 124–126 °C; ^1^H NMR (400 MHz, DMSO-d_6_) *δ* 8.97 (s, 1H, Ar-H), 8.66 (bs, 1H, NH), 7.77 (s, 1H, Ar-H), 7.50 (d, *J* = 9.6 Hz, 1H, Ar-H), 7.34–7.29 (m, 5H, Ar-H), 6.91 (dd, *J* = 2.4 Hz, *J* = 8.8 Hz, 1H, Ar-H), 5.56 (s, 2H, CH_2_Ph), 3.79 (s, 3H, OCH_3_), 3.37–3.34 (m, 2H, C*H_2_*NH), 2.76–2.63 (m, 6H, CH_2_N, 2C*H_2_*CH_3_), 1.03 (t, *J* = 7.2 Hz, 6H, 2CH_2_C*H_3_*).

#### General procedure for the synthesis of 2-(1-benzyl-5-hydroxy-1H-indol-3-yl)-N-(dialkylaminoalkyl)glyoxylamides 14 and 15, 2–(1-benzyl-5-hydroxy-1H-indol-3-yl)-N-(hydroxyalkyl)glyoxamides 20 and 21 and ethyl 1-benzyl-5-hydroxy-1H-indole-3-carboxylate 29

Boron tribromide (1.0 M in methylene chloride, 0.51 ml, 3.0 mmol) was added dropwise, under a nitrogen atmosphere, to a solution of the appropriate derivative **12**, **13**, **18**, **19** or **28** (0.30 mmol) in anhydrous DCM, cooled at −10 °C. The mixture was left under stirring for 30 min at −10 °C and subsequently at room temperature overnight. The solvent was removed under reduced pressure, and the residue was washed twice with methanol to hydrolyse the excess of BBr_3_. The residues obtained were purified by flash chromatography (DCM/MeOH in ratio 9.5:0.5 for **14**, **15**, **20** and **21**, EtOAc/petroleum ether 40–60 °C in ratio 5:5 for **29** as eluting system).

##### 2-(1-Benzyl-5-hydroxy-1H-indol-3-yl)-N-(3-(dimethylamino)propyl)glyoxylamide (14)

Yield: 42%; oil; ^1^H NMR (400 MHz, DMSO-d_6_) *δ* 9.25 (bs, 1H, OH), 8.87–8.85 (m, 2H, NH, Ar-H), 7.67 (d, *J* = 2.0 Hz, 1H, Ar-H), 7.39–7.27 (m, 6H, Ar-H), 6.75 (dd, *J* = 2.4 Hz, *J* = 8.8 Hz, 1H, Ar-H), 5.51 (s, 2H, CH_2_Ph), 3.30–3.25 (m, 2H, C*H_2_*NH), 3.07–3.03 (m, 2H, CH_2_N), 2.74 (s, 6H, 2CH_3_), 1.91–1.87 (m, 2H, CH_2_C*H_2_*CH_2_).

##### 2-(1-Benzyl-5-hydroxy-1H-indol-3-yl)-N-(2-(diethylamino)ethyl)glyoxylamide (15)

Yield: 70%; mp = 176–178 °C; ^1^H NMR (400 MHz, DMSO-d_6_) *δ* 9.25 (s, 1H, OH), 8.91 (s, 1H, Ar-H), 8.71 (bs, 1H, NH), 7.68 (d, *J* = 2.4 Hz, 1H, Ar-H), 7.40–7.28 (m, 6H, Ar-H), 6.75 (dd, *J* = 2.4 Hz, *J* = 8.8 Hz, 1H, Ar-H), 5.52 (s, 2H, CH_2_Ph), 3.40–3.37 (m, 2H, C*H_2_*NH), 2.78–2.77 (m, 6H, CH_2_N, 2C*H_2_*CH_3_), 1.05 (t, *J* = 7.2 Hz, 6H, 2CH_2_C*H_3_*).

##### 2-(1-Benzyl-5-hydroxy-1H-indol-3-yl)-N-(3-hydroxypropyl)glyoxamide (20)

Yield: 30%; mp = 169–171 °C; ^1^H NMR (400 MHz, DMSO-d_6_) *δ* 9.24 (bs, 1H, OH), 8.85 (s, 1H, Ar-H), 8.67 (t, *J* = 6.4 Hz, 1H, NH), 7.66 (d, *J* = 2.4 Hz, 1H, Ar-H), 7.38–7.28 (m, 6H, Ar-H), 6.73 (dd, *J* = 2.4 Hz, *J* = 8.8 Hz, 1H, Ar-H), 5.50 (s, 2H, CH_2_Ph), 4.51 (t, *J* = 5.2 Hz, 1H, CH_2_O*H*), 3.49–3.44 (m, 2H, C*H*_2_OH), 3.30–3.26 (m, 2H, C*H*_2_NH), 1.69–1.65 (m, 2H, CH_2_C*H*_2_CH_2_).

##### 2-(1-Benzyl-5-hydroxy-1H-indol-3-yl)-N-(2-hydroxyethyl)glyoxamide (21)

Yield: 67%; mp = 217–219 °C; ^1^H NMR (400 MHz, DMSO-d_6_) *δ* 9.25 (s, 1H, OH), 8.88 (s, 1H, Ar-H), 8.58 (t, *J* = 5.8 Hz, 1H, NH), 7.67 (d, *J* = 2.4 Hz, 1H, Ar-H), 7.38–7.28 (m, 6H, Ar-H), 6.73 (dd, *J* = 2.4 Hz, *J* = 8.8 Hz, 1H, Ar-H), 5.51 (s, 2H, CH_2_Ph), 4.79 (t, *J* = 5.6 Hz, 1H, CH_2_O*H*), 3.53–3.49 (m, 2H, C*H*_2_OH), 3.31–3.26 (m, 2H, C*H*_2_NH).

##### Ethyl 1-benzyl-5-hydroxy-1H-indole-3-carboxylate (29)

Yield: 54%; mp = 175–177 °C; ^1^H NMR (400 MHz, DMSO-d_6_) *δ* 9.08 (s, 1H, OH), 8.16 (s, 1H, Ar-H), 7.39 (d, *J* = 2.0 Hz, 1H, Ar-H), 7.35–7.25 (m, 6H, Ar-H), 6.68 (dd, *J* = 2.4 Hz, *J* = 8.8 Hz, 1H, Ar-H), 5.42 (s, 2H, CH_2_Ph), 4.26 (q, *J* = 7.2 Hz, 2H, OC*H*_2_CH_3_), 1.33 (t, *J* = 7.2 Hz, 3H, OCH_2_C*H*_3_).

#### General procedure for the synthesis of 2–(1-benzyl-5-acetoxyethoxy-1H-indol-3-yl)-N-(dialkylaminoalkyl)glyoxylamides 16 and 17

To a solution of the appropriate derivative **14** or **15** (0.26 mmol) in acetone (12 ml), Cs_2_CO_3_ (261 mg, 0.80 mmol) and 2-bromoethyl acetate (0.044 ml, 0.40 mmol) were added. The mixture was stirred at 60 °C overnight, concentrated in vacuum and the residue suspended in ethyl acetate. The suspension was washed with H_2_O, saturated aqueous NaHCO_3_ solution and brine. The organic phase was dried over MgSO_4_ and concentrated under reduced pressure. The crude products were then purified by flash chromatography (DCM/MeOH in ratio 9:1 as eluting system).

##### 2-(1-Benzyl-5-acetoxyethoxy-1H-indol-3-yl)-N-(3-(dimethylamino)propyl)glyoxylamide (16)

Yield: 42%; mp = 97–99 °C; ^1^H NMR (400 MHz, CDCl_3_) *δ* 9.03 (s, 1H, Ar-H), 8.18 (t, *J* = 5.6 Hz, 1H, NH), 7.97 (d, *J* = 2.4 Hz, 1H, Ar-H), 7.37–7.29 (m, 3H, Ar-H), 7.21–7.18 (m, 3H, Ar-H), 6.94 (dd, *J* = 2.4 Hz, *J* = 8.8 Hz, 1H, Ar-H), 5.36 (s, 2H, CH_2_Ph), 4.47 (t, *J* = 4.8 Hz, 2H, CH_3_COOC*H_2_*), 4.29 (t, *J* = 4.8 Hz, 2H, OCH_2_), 3.52–3.48 (m, 2H, C*H*_2_NH), 2.64 (t, *J* = 7.2 Hz, 2H, CH_2_N), 2.45 (s, 6H, 2NCH_3_), 2.13 (s, 3H, CH_3_CO), 1.96–1.91 (m, 2H, CH_2_C*H_2_*CH_2_).

##### 2-(1-Benzyl-5-acetoxyethoxy-1H-indol-3-yl)-N-(2-(diethylamino)ethyl)glyoxylamide (17)

Yield: 60%; oil; ^1^H NMR (400 MHz, CDCl_3_) *δ* 9.04 (s, 1H, Ar-H), 8.02 (bs, 1H, NH), 7.98 (d, *J* = 2.4 Hz, 1H, Ar-H), 7.34–7.29 (m, 3H, Ar-H), 7.19–7.16 (m, 3H, Ar-H), 6.92 (dd, *J* = 2.4 Hz, *J* = 8.8 Hz, 1H, Ar-H), 5.35 (s, 2H, CH_2_Ph), 4.45 (t, *J* = 4.6 Hz, 2H, CH_3_COOC*H_2_*), 4.28 (t, *J* = 4.6 Hz, 2H, OCH_2_), 3.48–3.44 (m, 2H, C*H_2_*NH), 2.69 (t, *J* = 6.2 Hz, 2H, CH_2_N), 2.62 (q, *J* = 3.6 Hz, 4H, 2C*H_2_*CH_3_), 2.11 (s, 3H, CH_3_CO), 1.09 (t, *J* = 7.2 Hz, 6H, 2CH_2_C*H_3_*).

#### General procedure for the synthesis of 2-(1-benzyl-5-methoxy-1H-indol-3-yl)-N-(hydroxyalkyl)glyoxamides 18 and 19

To a solution of compound **11** (983 mg, 3.0 mmol) in 1,4-dioxane (3 ml), the appropriate amine (3-amino-1-propanol for **18** or ethanolamine for **19**, 9.0 mmol) was added dropwise at 0 °C and the mixture was stirred for 3 h at room temperature. The dioxane was removed under reduced pressure and water was added to the mixture; the generated precipitate was collected by vacuum filtration. The crude products were finally purified by flash chromatography (EtOAc/petroleum ether 40–60 °C in ratio 7:3 as eluting system).

##### 2-(1-Benzyl-5-methoxy-1H-indol-3-yl)-N-(3-hydroxypropyl)glyoxamide (18)

Yield: 71%; mp = 125–127 °C; ^1^H NMR (400 MHz, CDCl_3_) *δ* 9.05 (s, 1H, Ar-H), 7.95 (d, *J* = 2.4 Hz, 1H, Ar-H), 7.85 (t, *J* = 6.0 Hz, 1H, NH), 7.37–7.32 (m, 3H, Ar-H), 7.20–7.18 (m, 3H, Ar-H), 6.91 (dd, *J* = 2.8 Hz, *J* = 9.2 Hz, 1H, Ar-H), 5.37 (s, 2H, CH_2_Ph), 3.91 (s, 3H, OCH_3_), 3.72 (t, *J* = 8.0 Hz, 2H, C*H_2_*OH), 3.61–3.55 (m, 2H, C*H_2_*NH), 1.86–1.81 (m, 2H, CH_2_C*H_2_*CH_2_).

##### 2-(1-Benzyl-5-methoxy-1H-indol-3-yl)-N-(2-hydroxyethyl)glyoxamide (19)

Yield: 35%; mp = 154–156 °C; ^1^H NMR (400 MHz, DMSO-d_6_) *δ* 8.94 (s, 1H, Ar-H), 8.61 (t, *J* = 6.0 Hz, 1H, NH), 7.77 (s, 1H, Ar-H), 7.49 (d, *J* = 9.2 Hz, 1H, Ar-H), 7.37–7.28 (m, 5H, Ar-H), 6.91 (dd, *J* = 2.4 Hz, *J* = 8.8 Hz, 1H, Ar-H), 5.56 (s, 2H, CH_2_Ph), 4.79 (bs, 1H, OH), 3.79 (s, 3H, OCH_3_), 3.52–3.51 (m, 2H, C*H_2_*OH), 3.32–3.29 (m, 2H, C*H_2_*NH).

#### General procedure for the synthesis of 5-(dialkylaminoalkoxy)-1-benzyl-1H-indole-3-carboxylic acids 23–25

LiOH·H_2_O (21 mg, 0.49 mmol) was added to an ice-cooled solution of the appropriate derivative **8**, **9**, or **22** (0.81 mmol) in 8 ml of MeOH/H_2_O mixture (3:1). The resulting mixture was refluxed overnight. The suspension was filtered off, and the filtrate was acidified with a 10% aqueous solution of HCl until pH = 1. The mixture was extracted with DCM and, after drying with MgSO_4_, the solvent was evaporated to dryness to yield the crude products **23–25**, which did not need any further purification.

##### 5-(3-(Dimethylamino)propoxy)-1-benzyl-1H-indole-3-carboxylic acid (23)

Yield: 97%, oil, ^1^H NMR (400 MHz, MeOD) *δ* 8.00 (s, 1H, Ar-H), 7.64 (d, *J* = 2.4 Hz, 1H, Ar-H), 7.32–7.29 (m, 4H, Ar-H), 7.27–7.19 (m, 2H, Ar-H), 6.87 (dd, *J* = 2.4 Hz, *J* = 9.2 Hz, 1H, Ar-H), 5.41 (s, 2H, CH_2_Ph), 4.16 (t, *J* = 5.8 Hz, 2H, OCH_2_), 3.38 (t, *J* = 7.6 Hz, 2H, CH_2_N), 2.94 (s, 6H, 2CH_3_), 2.25–2.21 (m, 2H, CH_2_C*H_2_*CH_2_).

##### 5-(2-(Dimethylamino)ethoxy)-1-benzyl-1H-indole-3-carboxylic acid (24)

Yield: 81%; mp = 130–132 °C; ^1^H NMR (400 MHz, MeOD) *δ* 8.06 (s, 1H, Ar-H), 7.73 (d, *J* = 2.0 Hz, 1H, Ar-H), 7.37–7.27 (m, 4H, Ar-H), 7.23–7.21 (m, 2H, Ar-H), 6.97 (dd, *J* = 8.8 Hz, *J* = 2.0 Hz, 1H, Ar-H), 5.45 (s, 2H, CH_2_Ph), 4.41–4.40 (m, 2H, OCH_2_), 3.63–3.61 (m, 2H, CH_2_N), 3.01 (s, 6H, 2CH_3_).

##### 5-(2-(Diethylamino)ethoxy)-1-benzyl-1H-indole-3-carboxylic acid (25)

Yield: 90%; mp = 94–96 °C; ^1^H NMR (400 MHz, DMSO-d_6_) *δ* 8.21 (s, 1H, Ar-H), 7.57 (d, *J* = 2.4 Hz, 1H, Ar-H); 7.47 (d, *J* = 9.2 Hz, 1H, Ar-H); 7.35–7.29 (m, 2H, Ar-H); 7.27–7.25 (m, 3H, Ar-H); 6.90 (dd, *J* = 2.4 Hz, *J* = 9.2 Hz, 1H, Ar-H); 5.48 (s, 2H, CH_2_Ph); 4.35 (t, *J* = 4.6 Hz, 2H, OCH_2_); 3.52–3.50 (m, 2H, CH_2_N); 3.23–3.21 (m, 4H, 2C*H_2_*CH_3_); 1.25 (t, *J* = 7.2 Hz, 6H, 2CH_2_C*H_3_*).

##### Ethyl 5-methoxy-1H-indole-3-carboxylate (27)

To a solution of compound **26** (574 mg, 3.00 mmol) in absolute ethanol (52 ml) a catalytic amount of concentrated sulphuric acid (0.52 ml) was added, and the resulting mixture was allowed to reflux for 20 h. After completion of the reaction, ethanol was removed, and the residue was dissolved with ethyl acetate and washed with saturated NaHCO_3_ solution. The organic layer was dried over MgSO_4_, filtered, and evaporated to give the corresponding ester. The crude product was then purified by flash chromatography (EtOAc/petroleum ether 40–60 °C in ratio 5:5 as eluting system). Yield: 31%; mp = 123–125 °C; ^1^H NMR (400 MHz, CDCl_3_) *δ* 8.56 (bs, 1H, NH), 7.89 (d, *J* = 3.2 Hz, 1H, Ar-H), 7.70 (d, *J* = 2.8 Hz, 1H, Ar-H), 7.32 (d, *J* = 8.8 Hz, 1H, Ar-H), 6.93 (dd, *J* = 8.8 Hz, *J* = 2.4 Hz, 1H, Ar-H), 4.41 (q, *J* = 7.2 Hz, 2H, OC*H_2_*CH_3_), 3.91 (s, 3H, OCH_3_), 1.45 (t, *J* = 7.0 Hz, 3H, OCH_2_C*H_3_*).

##### Ethyl 1-benzyl-5-methoxy-1H-indole-3-carboxylate (28)

Sodium hydride (40 mg, 1.00 mmol, 60% dispersion in mineral oil) was added portionwise, under nitrogen atmosphere, to an ice-cold solution of compound **27** (200 mg, 0.91 mmol) in 5 ml of DMF and the mixture was stirred for about 30 min at 0 °C. Once hydrogen evolution ceased, benzyl bromide (0.12 ml, 1.00 mmol) was added dropwise, and the reaction was maintained under stirring for 5 h at room temperature. After completion of the reaction, DMF was evaporated under reduced pressure, then ice was added to the flask and the solid precipitate formed was collected by vacuum filtration. The crude product was then purified by flash chromatography (EtOAc – petroleum ether 40–60 °C in ratio 3:7 as eluting system). Yield: 83%; mp = 89–91 °C; ^1^H NMR (400 MHz, DMSO-d_6_) *δ* 8.24 (s, 1H, Ar-H), 7.50 (d, *J* = 2.4 Hz, 1H, Ar-H), 7.44 (d, *J* = 9.2 Hz, 1H, Ar-H), 7.35–7.25 (m, 5H, Ar-H), 6.84 (dd, *J* = 2.4 Hz, *J* = 8.8 Hz, 1H, Ar-H), 5.47 (s, 2H, CH_2_Ph), 4.28 (q, *J* = 7.0 Hz, 2H, OC*H_2_*CH_3_), 3.78 (s, 3H, OCH_3_), 1.34 (t, *J* = 7.0 Hz, 3H, OCH_2_C*H_3_*).

#### Carbonic anhydrases activation assays

A stopped-flow method^38^ has been used for assaying the CA catalysed CO_2_ hydration activity with Phenol red as indicator, working at the absorbance maximum of 557 nm, following the initial rates of the CA-catalysed CO_2_ hydration reaction for 10–100 s. For each activator, at least six traces of the initial 5–10% of the reaction have been used for determining the initial velocity. The uncatalyzed rates were determined in the same manner and subtracted from the total observed rates. Stock solutions of activator (0.1 mM) were prepared in distilled-deionized water and dilutions up to 0.1 nM were done thereafter with the assay buffer. The activation constant (K_A_), defined similarly with the inhibition constant (K_I_), was obtained by considering the classical Michaelis–Menten equation (Equation (3), which has been fitted by nonlinear least squares by using PRISM 3:

$$v=v_{\text{max}}/\left\{1+K_{M}/\left[S\right]\left(1+{\left[A\right]}_{f}/K_{A}\right)\right\}$$

(3)


where [A]_f_ is the free concentration of activator.

Working at substrate concentrations considerably lower than K_M_ ([S]≪K_M_), and considering that [A]_f_ can be represented in the form of the total concentration of the enzyme ([E]_t_) and activator ([A]_t_), the obtained competitive steady-state equation for determining the activation constant is given by Equation 4:

$$v=v_{0}K_{A}/\{K_{A}+{\left[A\right]}_{t}–0.5\{\left({\left[A\right]}_{t}+{\left[E\right]}_{t}+{\text{ K}}_{A}\right)–{\left({\left[A\right]}_{t}+{\left[E\right]}_{t}+{\text{ K}}_{A}\right)}^{2}–4{\left[A\right]}_{t}{\left[E\right]}_{t}{}^{1}/2\}\}$$

(4)


where v_0_ represents the initial velocity of the enzyme-catalysed reaction in the absence of an activator^39–42^. Enzyme concentrations in the assay system were in the range of 6.5–12.0 nM.

#### Molecular modeling

To perform the molecular modelling studies on the newly discovered CAAs, the latest version of docking software i.e. AutodockGPU^43^ along with its Graphical User Interface AutoDockTools (ADT)^44^ were employed. In this study, we attempted to mimic the proposed two-step reversible process. This included the simulation of the ligand-protein complex before and after the proton shuttling has taken place. In this attempt, to mimic the first step, the X-ray crystal structure of hCA VII having the (PDB ID-3MDZ)^45^, solved at 2.32Å resolution, was downloaded from the RCSB PDB database^46^. The zinc-bound water molecule from hCA II (PDB ID-1AVN)^18^ was retained and placed into the hCA VII structure. The preparation of the protein structure was performed using the protein preparation wizard of Maestro Suite^47^^,^^48^. This routine adds the hydrogen atoms and bond orders to produce suitable protonation states; also all the water molecules were deleted apart from the one that coordinates the zinc. Before docking, the co-crystal ligand was also separated from the 3MDZ protein. To mimic the second step (namely after the proton shuttling event), the same X-ray structure of hCA VII-3MDZ was utilised and the H_2_O bound to Zn^2+^ converted to OH^-^. The most active compound **7** was built into its protonated and neutral form using the 2 D sketcher of Maestro and the optimised geometry of the ligand was obtained using the “minimize ligand” option of the same Maestro suite. Via Maestro, the X-ray crystal structures of hCA I (PDB ID-6EVR)^49^, hCA II (PDB ID-3K34)^50^, were also retrieved and superimposed on the hCA VII coordinates. Both the protein and ligands were translated in the AD4 format (PDBQT) using the python scripts prepare_ligand4.py and prepare_receptor4.py, part of ADT, applying the standard settings. From the literature survey, it is a well-known fact that the CA activators do not bind the zinc but bind at the entry site far away from the Zn^2+^ ion^7^^,^^51^. For the aforementioned docking, the crystal structure of hCA II bound with the activator of histamine was retrieved and superimposed on the hCA VII and the grid was set at the centre of the histamine molecule. A set of grids of 50 Å × 50 Å × 50 Å with 0.375 Å spacing was calculated considering the docking area for all the ligand atom types utilising the AutoGrid4 module. 100 independent dockings were accomplished. Each docking calculation included 20 million energy evaluations using the Lamarckian Genetic Algorithm Local Search (GALS) method. This method results in a population of feasible docking solutions and gives the best discrete generation of a binding pose. According to the technique of Solis and Wets, a low-frequency local search method was applied to every docking attempt to make sure that the representation of the final solution is a local minimum. In our docking protocol, 300 iterations of Solis and Wets local search were applied with a probability of 0.06 and a population size of 250. To generate new docking attempts for subsequent generations, a mutation rate of 0.02 and a crossover rate of 0.8 were used, and the best individual from each generation was propagated over the following generation. The docking results from each of the 100 independent docking calculations were clustered based on the root-mean-square deviation (rmsd) between the cartesian coordinates of the atoms (solutions varying by less than 2.0) and scored based on the calculated free energy of binding (ΔG_AD4_). Using the calculated ΔG_AD4_ and docking cluster population size, the best binding poses of **7**, both before and after the proton shuttling step, were chosen among the 100 independent conformations/configuration states resulting from docking experiments.

#### Biological assay

##### Human microglial cell line

All materials for cell culturing were obtained by Corning, New York, USA, and all used reagents were purchased by Sigma-Aldrich (Saint Louis, MO, USA). The human microglial cell line C20, originally generated by David Alvarez-Carbonell et al.^52^, was grown in DMEM-F12 medium supplemented with 10% of FBS, 100 U/mL penicillin, 0.1 mg/mL streptomycin, and neomycin (600 μg/mL), as a selector of the immortalised telomerase-expressing cells. Cells were kept at 37 °C under a humidified atmosphere with 95% O_2_ and 5% CO_2_. Viable cells (identified by counting trypan-blue-excluding cellular elements) were plated at a density of 100,000 cells each well in a 24-well plate. To evaluate the ability of target compounds to modulate the cellular viability and brain-derived neurotrophic factor (BDNF) release, C20 cells were challenged with CAAs.

##### Cell viability assay

C20 cells were seeded in 96-well microplates at a density of 5000 cells/well and maintained in complete culture media. The day after the seed, cells were treated with CAAs **7**, **8**, and **9** for 24 h (1 and 10 µM). The cell viability was determined using the [3–(4,5-dimethylthiazol-2-yl)-5–(3-carboxymethoxyphenol)-2–(4-sulfophenyl)-2H-tetrazolium, inner salt] (MTS) assay according to the manufacturer’s instructions (Promega, Milano, Italy). This tetrazolium dye can be reduced by the metabolic reducing agents NADH and NADPH to a water-soluble formazan salt. The amount of formazan produced is considered to be a marker of cell viability. The MTS reagent was added to treated C20 cells and the colorimetric conversion of formazane was quantified after 1 h by measuring the absorbance at 490 nm (EnSightTM multimode plate reader, equipped with Kaleido Data Acquisition and Analysis Software).

##### BDNF enzyme-linked immunosorbent assay

C20 cells were seeded in 24-well microplates at a density of 100,000 cells/well and maintained in complete culture media. The day after the seed, cells were challenged with compound **7** for 2 h in serum-free medium and then maintained in fresh serum-free medium for 22 h. At the end of the 24 h, the levels of BDNF were measured in the C20 conditioned medium using an enzyme-linked immunosorbent assay (ELISA) Kit (SEA011Mi 96 Tests, Cloude-Clone Corp., CCC, USA), accordingly to manufacture instructions. Briefly, the cell culture supernatants were centrifuged for 20 min at 1000 × g, and used in the ELISA assay. The microplate provided in the kit has been pre-coated with an antibody specific for BDNF. Standards or samples were added to the microplate wells and incubated for 1 h at 37 °C. Next, a biotin-conjugated antibody specific to BDNF was added to the well for 1 h. After washing, avidin conjugated to Horseradish Peroxidase was added to each microplate well and following incubation and washing, TMB substrate solution was added. The enzyme-substrate reaction was terminated by the addition of sulphuric acid solution and the colour change was measured spectrophotometrically at a wavelength of 450 nm ± 10 nm. The concentration of BDNF in the samples was then determined by comparing the O.D. of the samples to the standard curve. Levels of BDNF were normalised on the number of cells counted in each well by crystal violet staining, and BDNF levels were reported as pg/mL normalised per total cells.

##### Data analysis

Values are presented as mean ± standard error of the mean (SEM) of 3 experiments. Either a Student’s *t*-test for independent means or one way ANOVA following by Bonferroni’s post-test were used to define statistical differences between absolute values, which were considered significant at *p* < 0.05.

## Results and discussion

### Chemistry

**Scheme 1.**
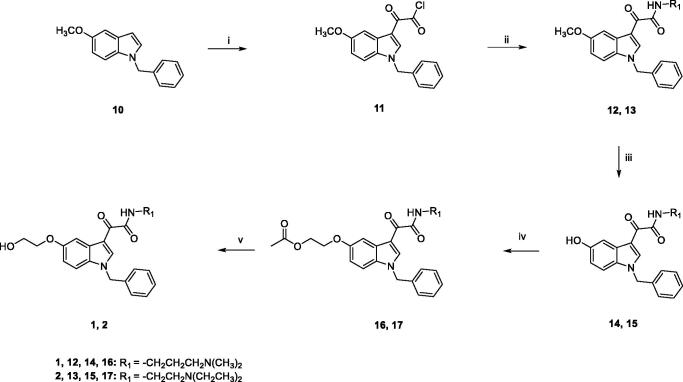
Reagents and conditions: (i) anhydrous Et_2_O, oxalyl chloride, room temperature, 2h; (ii) anhydrous toluene, *N*,*N*-dimethyl-1,3-propylenediamine (for **12**) or *N*,*N*-diethylethylenediamine (for **13**), NEt_3_, room temperature, overnight; iii) a) anhydrous DCM, BBr_3_, -10 °C, 30 min.; (b) room temperature, overnight; (iv) acetone, Cs_2_CO_3_, 2-bromoethylacetate, reflux, overnight; (v) 1,4-dioxane, 1M NaOH, room temperature, 5h.

Target compounds **1** and **2** were prepared according to the experimental procedure outlined in Scheme 1. Acylation of *N*-benzyl-5-methoxyindole **10** with oxalyl chloride, in anhydrous diethyl ether, at room temperature, yielded the corresponding indolylglyoxylyl chloride **11**, which was allowed to react with the appropriate amine (*N*,*N*-dimethyl-1,3-propylenediamine for **12**, *N*,*N*-diethylethylenediamine for **13**), in the presence of triethylamine, in dry toluene solution, at room temperature, to give compounds **12** and **13**. Compounds **14** and **15** were obtained by demethylation of **12** and **13**, using boron tribromide in anhydrous DCM at −10 °C for 30 min and then at room temperature overnight. Following treatment with 2-bromoethylacetate and Cs_2_CO_3_ in acetone at reflux overnight, led to compounds **16** and **17**, which were finally hydrolysed with 1 M NaOH solution in 1,4-dioxane at room temperature for 5 h, to achieve compounds **1** and **2**.

**Scheme 2.**
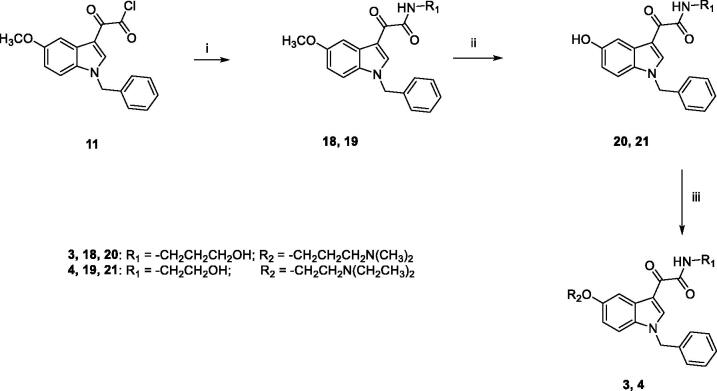
Reagents and conditions: (i) 1,4-dioxane, 3-amino-1-propanol (for **18**) or ethanolamine (for **19**), room temperature, 3 h; (ii) (a) anhydrous DCM, BBr_3_, −10 °C, 30 min.; (b) room temperature, overnight; (iii) anhydrous DMF, Cs_2_CO_3_, 3-dimethylamino-1-propylchloride hydrochloride (for **3**) or 2-chloro-*N*,*N*-diethylethylamine hydrochloride (for **4**), 60 °C, 5h.

The general synthetic pathway for the target compounds **3** and **4** is outlined in Scheme 2. Condensation of **11** with the appropriate amine (3-amino-1-propanol for **18**, ethanolamine for **19**) in 1,4-dioxane, at room temperature, yielded the amides **18** and **19**, which were then demethylated by treatment with boron tribromide in dry DCM. Subsequent reaction of **20** and **21** with the appropriate chloride (3-dimethylamino-1-propylchloride hydrochloride for **3**, 2-chloro-*N,N*-diethylethylamine hydrochloride for **4**) and Cs_2_CO_3_ in DMF for 5 h at 60 °C furnished the target compounds **3** and **4**, finally purified by flash chromatography.

**Scheme 3.**
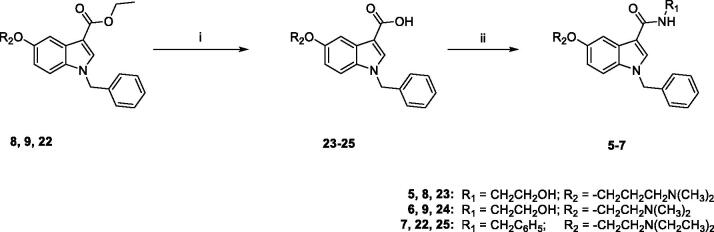
Reagents and conditions: (i) MeOH/H_2_O, LiOH H_2_O, 80 °C, overnight; (ii) anhydrous DMF, TBTU, DIPEA, ethanolamine (for **5** and **6**) or benzylamine (for **7**), room temperature, overnight.

**Scheme 4.**
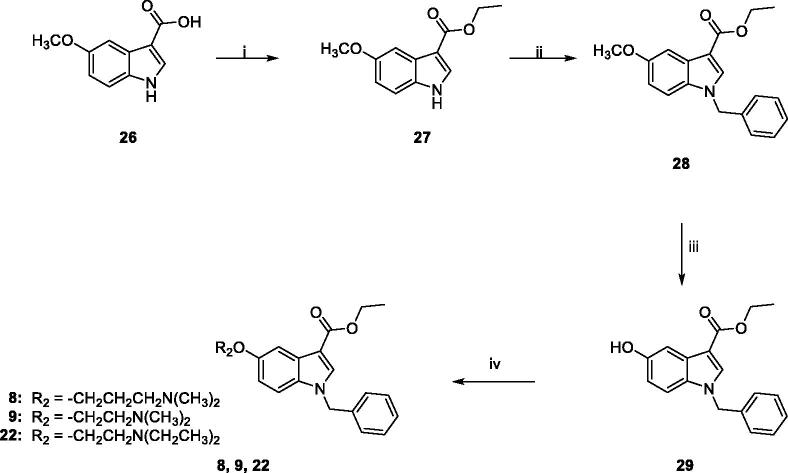
Reagents and conditions: (i) abs. EtOH, conc. H_2_SO_4_, 80 °C, 20 h; (ii) (a) NaH, dry DMF, 0 °C, 30 min.; (b) BzBr, room temperature, 5 h; (iii) (a) anhydrous DCM, BBr_3_, -10 °C, 30 min.; (b) room temperature, overnight; (iv) anhydrous DMF, Cs_2_CO_3_, 3-dimethylamino-1-propylchloride hydrochloride (for **8**) or 2-chloro-*N*,*N*-dimethylethylamine hydrochloride (for **9**) or 2-chloro-*N*,*N*-diethylethylamine hydrochloride (for **22**) or 60 °C, 5h.

The synthesis of target compounds **5–7** was carried out following the procedure reported in Scheme 3. The ethylester derivatives **8**, **9**, **22**, prepared as outlined in Scheme 4, were hydrolysed with LiOH·H_2_O in a solution of MeOH/H_2_O (3:1), to obtain **23–25**, which were then treated with 2-(*1H*-benzotriazole-1-yl)-1,1,3,3-tetramethyluronium tetrafluoroborate (TBTU) as condensing agent, *N,N-*diisopropylethylamine (DIPEA) and the appropriate amine (ethanolamine for **5** and **6**, benzylamine for **7**) in anhydrous DMF, at room temperature overnight, to yield compounds **5–7**, finally purified by flash chromatography.

Scheme 4 outlines the synthetic procedure for the obtainment of derivatives **8**, **9**, **22**. The ethyl ester derivative **27** was obtained by refluxing the commercially available 5-methoxyindole-3-carboxylic acid **26** in absolute ethanol for 20 h, in the presence of a catalytic amount of concentrated sulphuric acid. Benzylation of **27** by treatment with benzyl bromide in dry DMF in the presence of NaH, and subsequent demethylation with boron tribromide in anhydrous DCM yielded **29**, which was finally reacted with the appropriate chloride (3-dimethylamino-1-propylchloride hydrochloride for **8**, 2-chloro-*N*,*N*-dimethylethylamine hydrochloride for **9**, 2-chloro-*N*,*N*-diethylethylamine hydrochloride for **22**) in the presence of Cs_2_CO_3_ in dry DMF at 60 °C for 5 h, yielding **8**, **9**, **22**, finally purified by flash chromatography.

### CA activation assays

Compounds **1**–**9** were assayed for their ability to activate four catalytically active and physiologically relevant hCA isoforms expressed in the human brain, namely the cytosolic hCA I, II, and VII and the mitochondrial hCA VA.

The cytosolic and ubiquitous CA I is expressed in the motor neurons in the human spinal cord^53^. The physiologically dominant isoform CA II is located both in the choroid plexus and in oligodendrocytes, myelinated tracts, astrocytes, and myelin sheaths in the vertebrates’ brain^54^. Immunocytochemical experiments demonstrated that astrocytes and neurons express the mitochondrial CA VA suggesting that this isozyme has a cell-specific, physiological role in the nervous system^55^. CA VII showed comparable expression in the cortex, hippocampus, and thalamus and might be considered a brain-associated CA being absent in the majority of other tissues^56^.

CA activation data of the indole-based derivatives **1–9** are listed in Table 1. The following structure-activity relationships (SARs) for the activation of these enzymes can be drawn:

**Table 1.** CA activation of isoforms hCA I, II, VA, and VII with compounds **1**–9, by a stopped-flow CO2 hydrase assay. 
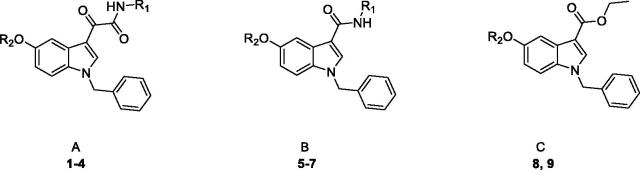

K_A_ (µM)^a^						
Cpd	R_1_	R_2_	hCA I [hCA VA/hCA VII]^b^	hCAII [hCA II/hCA VII]^b^	hCA VA [hCA VA/hCA VII]^b^	hCA VII
**1**	(CH_2_)_3_N(CH_3_)_2_	(CH_2_)_2_OH	>100 [>9.3]	>100 [>9.3]	25.0 [2.3]	10.8
**2**	(CH_2_)_2_N(CH_2_CH_3_)_2_	(CH_2_)_2_OH	88.9 [8.9]	>100 [>10.0]	28.7 [2.9]	10.0
**3**	(CH_2_)_3_OH	(CH_2_)_3_N(CH_3_)_2_	>100 [>9.4]	>100 [>9.4]	50.9 [4.8]	10.6
**4**	(CH_2_)_2_OH	(CH_2_)_2_N(CH_2_CH_3_)_2_	>100 [>11.0]	>100 [>11.0]	34.4 [3.8]	9.1
**5**	(CH_2_)_2_OH	(CH_2_)_3_N(CH_3_)_2_	>100 [>9.6]	>100 [>9.6]	28.0 [2.7]	10.4
**6**	(CH_2_)_2_OH	(CH_2_)_2_N(CH_3_)_2_	>100 [>9.3]	>100 [>9.3]	26.8 [2.5]	10.7
**7**	CH_2_C_6_H_5_	(CH_2_)_2_N(CH_2_CH_3_)_2_	>100 [>13.3]	>100 [>13.3]	24.4 [3.2]	7.5
**8**	–	(CH_2_)_3_N(CH_3_)_2_	69.1[9.6]	>100 [>13.9]	59.8 [8.3]	7.2
**9**	–	(CH_2_)_2_N(CH_3_)_2_	>100 [>12.2]	>100 [>12.2]	29.1 [3.5]	8.2
Histamine			2.1	125	0.010	37.5

^a^Mean from 3 different assays, by a stopped flow technique (errors were in the range of ±5–10% of the reported values). ^b^K_A_ ratio for the indicated enzyme isoforms.

- the cytosolic isoforms hCA I and II were not significantly activated by these derivatives up to concentrations as high as 100 µM, with the exception of compounds **2** and **8** which showed K_A_ values of 88.9 and 69.1 µM for hCA I, respectively.
- The mitochondrial isoform hCA VA was moderately activated by all derivatives with activation constants ranging between 24.4 and 59.8 µM; the best activators were **1**, **2**, **4**–**7**, **9**, whereas **3** and **8** showed a poor activity.
- The brain associated cytosolic CA isoform (hCA VII) was the most sensitive one to activation by all compounds, with K_A_ values in the range of 7.2–10.8 µM, independently from the decoration pattern of the central indole nucleus, suggesting that this good activity might be ascribed to the presence of the protonatable moiety rather than to its position.

Overall, these results pointed out compounds **7**, **8**, and **9** as the most interesting ones in terms of K_A_ values (7.5, 7.2, and 8.2, µM, respectively) and selectivity profile towards isoform VII, as they show selectivity ratios of >13.3–9.6 against hCAI, >13.9–>12.2 against hCAII, and 3.2–8.3 against hCA VA.

### Molecular docking studies and PK parameters prediction

**Figure 2.**
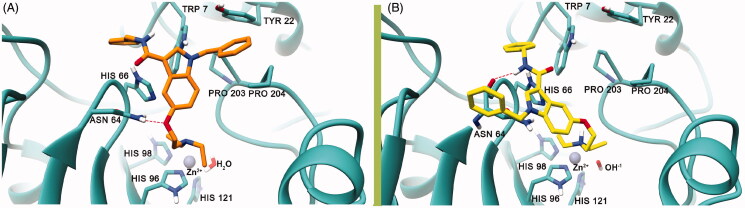
The best binding pose of **7** before (A) and after (B) the proton shuttling step in the hCA VII active site (PDB ID 3MDZ). The ligand is represented as orange (A) or yellow (B) sticks, while the protein structure is depicted in sea-green ribbons and sticks. Interacting residues are labelled. The Zn^2+^ ion is represented as a violet sphere. H-bonds are represented with the red dashed lines.

Molecular modelling studies were undertaken to suggest a theoretical model of the complex between the hCA VII and the newly discovered activators and provide a possible explanation for their modulating as well as selectivity properties. In particular, a sequence of in silico studies was executed on compound **7** that was found to be comparatively the most potent and selective hCA VII activator of the whole set. Docking calculations were performed using the AutoDockGPU software (see molecular modelling methods). Results of the docking studies (Figure 2(A)), attained to model the first step of chemical reaction (before the proton shuttling), revealed that the deprotonated diethylamine is lodged at a site far away from the Zn^2+^ ion and facing the His66 residue; this particular residue is said to be the most important residue in mediating the enzymatic activity of proton shuttle transfer reaction^57^. As per the previous studies reported by De Simone et al.^58^, Pinard et al.^59^, and West et al.^60^, the CA active site has an amphiphilic nature that has both a hydrophobic and a hydrophilic pocket next to the Zn^2+^ ion. The important proton shuttle mediating His66 residue falls into the hydrophilic region^59^^,^^60^. The ether oxygen at position 5 of the indole ring accepts an H-bond from the Asn64 side chain, while the indole moiety is involved in a π–π interaction with His66 residue. The *N*-benzyl ring attached to the indole scaffold is lodged into a cleft that is lined by several non-polar amino acids such as Pro203, Pro204 and is also participating to a parallel displaced π–π interaction with the Tyr22 residue that is present at the entry cleft of the active site. Similarly, the 3-carboxamidobenzyl group is engaging in a π–π interaction with the Trp7 side chain. Since docking calculations were only attained on **7**, we postulate that the other compounds reported here to activate hCA VII might establish the same interaction pattern also in light of the similar K_A_ experimental values (Table 1). Analysis of results attained by modelling the second step of the chemical reaction (i.e. after the proton shuttling) (Figure 2(B)) revealed that the protonated diethylamine and the 3-carboxamidobenzyl group is also lodged in the same protein region predicted to host the unprotonated ligand, forming a similar π–π interaction with Trp7 side chain; the 3-carboxamidobenzyl is also involved in the π–π interaction with His66, with the amide nitrogen donating an H-bond to the backbone CO of Asn64. In this position, the ligand protonated amine is also involved in a cation-π interaction with the His96. All in all, the position of the protonated and deprotonated amine of **7** might support the already postulated theory behind the activation mechanism through the proton shuttling process^14^.

**Figure 3.**
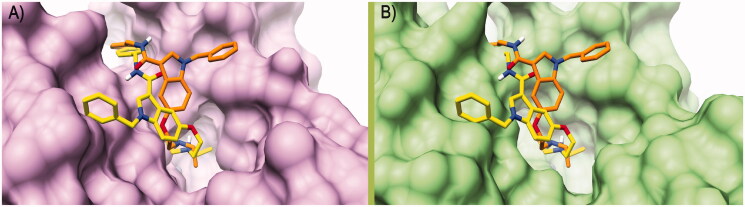
Binding poses calculated for compound **7** (orange and yellow) rigidly translated into the hCA I structure (PDB ID 6EVR, pink surface, A) and hCA II structure (PDB ID 3K34, green surface, B). All the pictures were rendered using UCSF Chimaera software^61^.

To rationalise the selectivity profile of **7**, the binding poses (protonated and neutral ligand) calculated into the hCA VII binding site were rigidly translated into the binding sites of hCA I and hCA II (Figure 3(A,B)). In this particular analysis, both the binding poses of **7** would give rise to multiple steric clashes in hCA I and hCA II isoforms. Most precisely, the neutral ligand’s diethylamine and the protonated ligand’s *N*-benzyl ring would induce clashes within the hCA I binding site (Figure 3(A)), whereas the steric hindrance of the 3-carboxamidobenzyl group would clash with the hCA II active site. All in all, these data would indicate that the orientation adopted by **7** into the hCA VII active site, favouring the proton shuttling step of the enzyme catalytic process, does not seem to be viable into the hCA I and II isoenzymes. Unfortunately, a similar analysis could not be attained for the hCA V due to the absence of the experimental atomic coordinates for this enzyme.

Given the possible implication of the CA activation for the treatment of CNS diseases, we were further asked if the most interesting compound **7** might also feature specific physicochemical parameters that could allow inferring a CNS activity. These studies were conducted in silico with the Qikprop program (Schrödinger. LLC New York (Table 2). From these studies, **7** should have a high potential of reaching the CNS. Indeed, the QPPMDCK parameter, which estimates the penetration of MDCK cells as a model of the blood-brain barrier (BBB), indicates that **7** should have high brain penetration properties. Also, this compound should be orally bioavailable considering its high calculated QPPCaco, which predicts the apparent Caco-2 permeability as a model of the gut-blood barrier.

**Table 2.** Calculated physicochemical and pharmacokinetic properties of **7**.

Parameter	7	Range or recommended values^a^
#rotor^b^	11.000	0.0/15.0
Mol MW^c^	455.599	130.0/725.0
Dipole^d^	6.972	1.0/12.5
DonorHB^e^	1.000	0.0/6.0
AccptHB^f^	5.250	2.0/20.0
QPlogPo/w^g^	6.455	−2.0/6.5
QPlogS^h^	−6.598	−6.5/0.5
QPPCaco^i^	1209.191	<25 Poor, >500 great
QPlogBB^j^	−0.076	−3.0/1.2
QPPMDCK^k^	672.043	<25 Poor, >500 great
QPlogKhsa^l^	1.261	−1.5/1.5
PercentHumanOralAbsorption^m^	100.000	(<25% is Poor)

^a^For 95% of known drugs. ^b^Number of non-trivial (not CX3), non-hindered (not alkene, amide, small ring) rotatable bonds. ^c^Molecular weight of the molecule. ^f^Computed dipole moment of the molecule. ^d^Computed dipole moment of the molecule. ^e^Estimated number of hydrogen bonds that would be donated by the solute to water molecules in an sueous solution. ^f^Estimated number of hydrogen bonds that would be accepted by the solute from water molecules in an aqueous solution. ^g^Predicted octanol/water partition coefficient. ^h^Predicted aqueous solubility, log S. S in mol dm^−3^ is the concentration of the solute in a saturated solution that is in equilibrium with the crystalline solid. ^i^Predicted apparent Caco-2 cell permeability in nm/sec. ^j^Predicted brain/blood partition coefficient. ^k^Predicted apparent MDCK cell permeability in nm/sec. MDCK cells are considered to be a good mimic for the blood-brain barrier. ^l^Prediction of binding to human serum albumin. ^m^Predicted qualitative human oral absorption.

### Effect of indole-based CCAs on BDNF level secreted by human microglial cells

To explore the neuroprotective potential of the newly synthesised indole-based CAAs, their capacity to promote brain-derived neurotrophic factor (BDNF) release was preliminarily tested on the C20 human microglial cell line. Microglial cells protect physiological brain function by regulating neurogenesis, neuronal survival, synaptic plasticity, and responding to alterations of the extracellular environment^62^. In fact, in the presence of a potentially dangerous signal, microglia rapidly undertake morphological and functional alterations (microglia activation) that may lead to the secretion of specific factors, including BDNF. This latter is one of the key neurotrophic factors responsible for neuronal survival and differentiation, and is also directly involved in the control of synaptic plasticity as a neuromodulator; its levels vary in different regions of the CNS during several neurological disorders, such as viral encephalitis, traumatic injury, ischaemia, multiple sclerosis and Parkinson’s disease^63–66^. Interestingly, a link between BDNF beneficial activities and CA activation has been previously demonstrated in neurons^61^^,^^67^, and correct acidification is a fundamental step during the release of BDNF from secretory granules^68^; this literature suggests a possible and strong relation between BDNF and CAs during neuroprotection. In light of this, microglia, taking an active part in neuroprotection by providing neurons with important survival factors, could represent a model of choice to study CAAs.

In this study, compounds **7**, **8**, and **9**, those with the highest activation towards the brain-related hCA VII isoform, were first tested for their cytotoxicity on microglial cells. To this end, human C20 cells were treated with the target compounds for 24 h with concentrations in the micromolar range, in line with K_A_ values, and then cell viability was evaluated by MTS assay.

**Figure 4.**
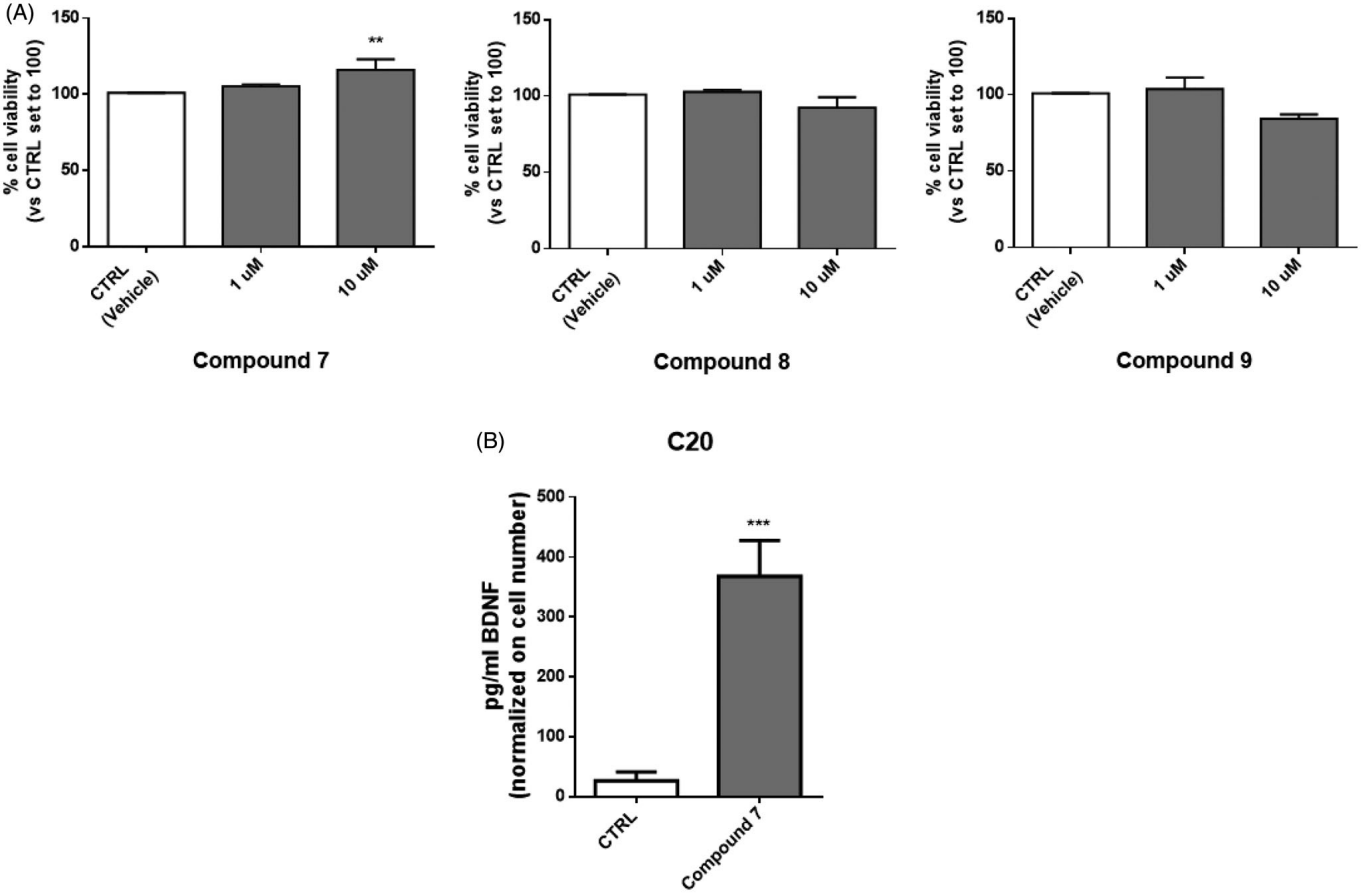
Treatment with compound **7** increases the level of BDNF released from human microglial cells. (A) C20 cells were exposed for 24 h at different concentrations of compounds **7**, **8** and **9**. Then the medium was replaced, and an MTS assay was performed to test cell viability. Results are reported as the percentage of the control group, treated with vehicle (DMSO). Data are represented as means ± SEMs of three independent experiments. The significance of the differences was determined by one-way ANOVA, which was followed by Bonferroni’s post-test: ** *p* < 0.01 vs. CTRL. B) The supernatant derived from C20 cells treated with 10 μM of compound **7** was collected and used for BDNF quantification by ELISA assay. Levels of BDNF (pg/ml) were then normalised on crystal violet absorbance. Data are represented as means ± SEMs of three independent experiments and the significance of the differences was determined by Student’s *t*-test analysis.

Compound **7** did not exhibit cytotoxic activity on C20 cells at any of the tested concentrations and even a little increase in cell viability was evidenced at 10 µM. On the contrary, compounds **8** and **9** caused a slight, although not statistically significant, reduction in cell viability at the highest tested concentration. These results prompted us to select compound **7** for further determination of its ability to promote the release of BDNF from human microglial cells. Human microglial cells were pre-treated for 2 h with compound **7**, then the medium was replaced and collected after 24 h for the evaluation of BDNF levels. Interestingly, in the medium derived from cells treated with compound **7** a significant increase in BDNF levels was evidenced, at amounts comparable to those reported in the literature for microglia (Figure 4)^69^.

These preliminary data would indicate that the neuroprotective potential of **7** could properly rely on the increased availability of extracellular BDNF, which in turn could reinforce the protective role on neurons, exerted by resting microglia towards potentially damaging events.

## Conclusions

In the present study three small series of indole-based derivatives were investigated for their activation profile on physiologically relevant human CA I, II, VA, and VII isoforms. Several compounds resulted as effective micromolar activators, with promising selectivity profiles towards the brain-associated cytosolic isoform hCA VII. Docking calculations provided a theoretical model explaining the CA activation profile of compound **7**, selected as the most potent and selective hCA VII activator of the whole set. Furthermore, in silico studies indicate that compound **7** possesses the physicochemical parameters suitable to reach the CNS and to be orally available. Finally, a preliminary biological evaluation showed its ability to increase the release of the brain-derived neurotrophic factor (BDNF) in microglial cells, combined with a lack of cytotoxicity. All in all these data highlighted **7** as a promising lead compound for the development of novel agents with potential application in the treatment of CNS-related diseases.
